# Mercury Exposure and Heart Diseases

**DOI:** 10.3390/ijerph14010074

**Published:** 2017-01-12

**Authors:** Giuseppe Genchi, Maria Stefania Sinicropi, Alessia Carocci, Graziantonio Lauria, Alessia Catalano

**Affiliations:** 1 Dipartimento di Farmacia e Scienze della Salute e della Nutrizione, Università della Calabria, 87036 Arcavacata di Rende (Cosenza), Italy; giuseppe.genchi@unical.it (G.G.); glauria@unical.it (G.L.); 2 Dipartimento di Farmacia-Scienze del Farmaco, Università degli Studi di Bari “A. Moro”, 70125 Bari, Italy; alessia.catalano@uniba.it

**Keywords:** mercury, antioxidants, cardiovascular diseases, cardiotoxicity, chelating agents

## Abstract

Environmental contamination has exposed humans to various metal agents, including mercury. It has been determined that mercury is not only harmful to the health of vulnerable populations such as pregnant women and children, but is also toxic to ordinary adults in various ways. For many years, mercury was used in a wide variety of human activities. Nowadays, the exposure to this metal from both natural and artificial sources is significantly increasing. Recent studies suggest that chronic exposure, even to low concentration levels of mercury, can cause cardiovascular, reproductive, and developmental toxicity, neurotoxicity, nephrotoxicity, immunotoxicity, and carcinogenicity. Possible biological effects of mercury, including the relationship between mercury toxicity and diseases of the cardiovascular system, such as hypertension, coronary heart disease, and myocardial infarction, are being studied. As heart rhythm and function are under autonomic nervous system control, it has been hypothesized that the neurotoxic effects of mercury might also impact cardiac autonomic function. Mercury exposure could have a long-lasting effect on cardiac parasympathetic activity and some evidence has shown that mercury exposure might affect heart rate variability, particularly early exposures in children. The mechanism by which mercury produces toxic effects on the cardiovascular system is not fully elucidated, but this mechanism is believed to involve an increase in oxidative stress. The exposure to mercury increases the production of free radicals, potentially because of the role of mercury in the Fenton reaction and a reduction in the activity of antioxidant enzymes, such as glutathione peroxidase. In this review we report an overview on the toxicity of mercury and focus our attention on the toxic effects on the cardiovascular system.

## 1. Introduction

As far back as 20,000–30,000 years BC, Paleolithic artists used various pigments, including cinnabar (mercuric sulfide, HgS) due to its red color, to draw hunting scenes with bison, bulls, stags, horses, humans, and handprints in negative images on cave walls (Altamira-Spain and Lascaux-France caves). Even the Chinese and the Romans (VII–VI century BC) employed cinnabar for pictorial art. Subsequently, mercury has been used in thermometers, sphygmomanometers, barometers, incandescent lights, and batteries; moreover, it was employed in dental amalgams (typically composed of 50% mercury, 25% silver, and 25% tin, copper, and nickel) [1], germicidal soaps, and skin creams [2]. Mercury has also been used to purify gold and silver minerals by forming amalgams in mines in the Brazil basin, in Laos, and in Venezuela. For a long time many medicines, cosmetics, and vaccines contained small amounts of organic mercury compounds, like ethylmercury thiosalicylate (thimerosal), as preservatives. Medicinal uses of mercury have included its use as a diuretic, antiseptic, skin ointment, laxative, and as a treatment of syphilis. Mercury has also been used as a poison. The great sculptor Benvenuto Cellini, when poisoned by a sublethal dose of mercury, was apparently cured of a severe case of syphilis [3]. History has left us a lot of information on the effect of mercury toxicity. The earliest recorded death by mercury is the Chinese Emperor Qin Shi Huang (260–210 BC). According to legend, the cause of death most likely was mercury poisoning, due to his immortality treatments [4]. Certainly, the exposure to mercury brought harmful effects to health of humans and called the attention of the scientific world after the epidemics occurred in Japan and in Iraq. In Japan, two methylmercury poisoning events are worthy of mention. These accidents, resulting from the deposition of industrial waste containing large quantities of methylmercury [5,6], occurred in the Japanese village of Minamata Bay (1953) and along the Agano river in Niigata (1964). Mercury, bioaccumulated within the food chain from plankton, microorganisms up to shellfish and fish, was then ingested, thus the inhabitants of Minamata Bay began to exhibit symptoms of neurological illness, such as uncontrollable trembling, loss of motor control, speech impairment, sensory disturbance, blindness, mental retardation, coma, and death. Infants, whose mothers were infected, developed mental retardation, peripheral neuropathy, and cerebral palsy. Additionally, in 1971 in rural Iraq, severe methylmercury intoxication occurred when bread was prepared and eaten from wheat seeds that had been treated with fungicides containing organic mercury compounds [6,7]. The incidents in Japan and Iraq produced not only deaths, but also multiple and long-lasting intoxication symptoms, including blindness, deafness, mental retardation, cerebral palsy, and dysarthria especially in children exposed in utero [8]. 

**Figure 1:**
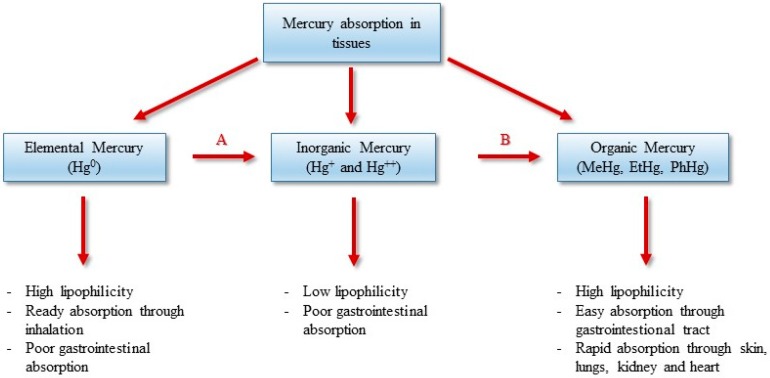
Bioavailabilty and toxic effects of mercury and its compounds. A: Oxidation in air, and enzymatically in red blood cells and tissues; B: Biomethylation by sulfate-reducing bacteria.

Methylmercury, the most toxic mercury compound, is an organic mercurial compound primarily found as a pollutant in rivers, lakes, and oceans. Methylmercury is usually formed naturally through biomethylation of mercury, carried out by aquatic anaerobic sulfate-reducing bacteria [9,10] (Figure 1). It also derives from anthropogenic sources, and when formed will be released into rivers, lakes, and oceans. Consequently, people whose diet consists mainly of shellfish and fish may be exposed to high levels of methylmercury [11]. Approximately 85% of methylmercury ingested is absorbed in the gastrointestinal tract, while about 5% is present in blood and 10% in the brain. In many developing countries, mercury is still a major problem which requires actions for proper control. Many efforts should be placed on the removal of anthropogenic sources of mercury and the prevention of exposure.

Several studies regarding mercury-related health problems have been carried out in populations mostly exposed through the consumption of mercury-contaminated fish and other seafood [12]. For decades, the toxic effects of mercury were associated mainly with the central nervous system. However, a growing body of evidence suggests that methylmercury exposure can also lead to increased risks of adverse cardiovascular impacts in exposed populations. In January 2010, the US eicosapentaenoic acid (EPA) assembled experts spanning epidemiology, toxicology, clinical medicine, risk, and exposure assessment to participate in a workshop in Washington DC to review the current science and literature concerning cardiovascular impacts of MeHg exposure. They studied MeHg exposure via fish, shellfish, and sea mammal consumption to elicit recommendations about whether these effects should be included in Hg regulatory impact analyses. The results of this workshop were reviewed by Roman et al. [13]. The authors assessed the causal relationship between MeHg exposure and an increased risk of cardiovascular health effects by evaluating the plausibility of biological mechanisms for the cardiovascular toxicity of MeHg and weighing the strength of the human, animal, and in vitro studies linking MeHg with cardiovascular health impacts. In this review, we attempt to present an understanding of the role that exposure to mercury plays in cardiovascular diseases.

## 2. Materials and Methods

The review was performed following the principles of the PRISMA statement [14]. A literature search of publications included in the electronic databases was conducted using MEDLINE (via PubMed) and Google Scholar. The search criteria considered the occurrence of the combination of the following keywords: mercury toxicity, heart disease, and cardiovascular disease either in the title, abstracts, or in the text. All the publications found were screened based upon consideration of the title and abstract in order to assess the relevance of the subject and eligibility. Each author independently extracted data from each paper regarding the role that exposure to mercury plays in cardiovascular diseases and discussed the data with the other authors. Then a draft of the manuscript was circulated to the authors and subsequently revised several times. A final version of the manuscript was then prepared and finally approved by all the authors.

## 3. Chemical Forms and Toxicity of Mercury

**Figure 2:**
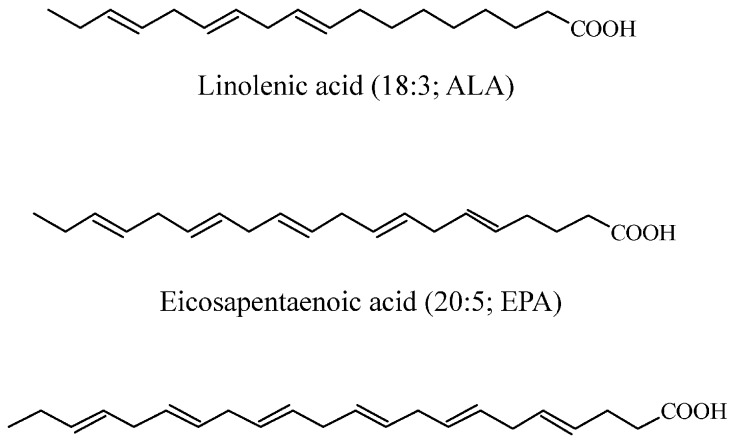
Main fatty acids of the omega-3 polyunsaturated fatty acids (PUFA) group.

Mercury (Hg, hydrargyrium from the Latin “liquid silver”) is a heavy metal (atomic number 80; atomic weight 200.59; density 13.59 g/cm^3^; melting point −39 °C; boiling point 359 °C) with a toxicity as well-known (World Health Organization 2007) [15] as lead and cadmium [16,17]. Mercury is a non-transition metal and is an extremely rare element in the Earth’s crust, usually in the form of the mineral cinnabar (mercury sulfide, HgS), having an average mass abundance of only 0.09 mg/Kg [18]. Mercury has three valence states and exists in several forms: inorganic mercury, which includes liquid metallic mercury and mercury vapor (Hg^0^), mercurous (Hg^+^) and mercuric (Hg^++^) salts, and organic mercury, with methylmercury (CH_3_Hg, MeHg), ethylmercury (C_2_H_5_Hg, EtHg), and phenylmercury (C_6_H_5_Hg, PhHg). The biological behavior and clinical significance of the various forms of mercury vary according to its chemical structure [19]. Elemental mercury (Hg^0^), at room temperature, exists in its liquid form which quickly turns to vapor when heated above room temperature. The high volatility of Hg^0^ prolongs the effects of anthropogenic release and Hg^0^ can remain suspended in the atmosphere for up to one year, where it can be transported and deposited globally. In the atmosphere, Hg^0^ constitutes the majority of mercury (>90%) and is the predominant form in the gaseous phase, which facilitates the long-range transport of mercury at a global scale [20]. Mercury is released into the environment from both natural and anthropogenic sources. Annually, volcanic (for example Etna and Stromboli, Sicily, Italy), geothermal outgassing activities (for example the Phlegrean Fields, Pozzuoli, Italy), thermal springs, earthquakes, erosion, and the volatilization of mercury present in the marine environment (Agency for Toxic Substances and Disease Registry, ATSDR 1999) [21,22,23] release an estimated 1500 t of mercury to the environment [24]. Anthropogenic release occurs from manifold industrial point sources, chlor-alkali plants [25] and coal-fired power plants [26] and is estimated to constitute 2320 t of mercury emitted annually into the atmosphere [24]. Hg^0^ is oxidized in air to its inorganic forms (Hg^+^ and Hg^++^) and is released during rain events to be deposited in soil and into the waters of rivers, lakes, and oceans. In its vapor form, metallic mercury is commonly absorbed through the respiratory tract, where it is poorly absorbed in the gastrointestinal tract. Because of its soluble characteristics, elemental mercury is highly diffusible through cell membranes as well as the blood-brain and placental barriers to reach target organs. Once in the blood stream, Hg^0^ is easily oxidized in red blood cells and tissues into inorganic Hg^+^ and Hg^++^ in the presence of catalase and peroxidase. The inorganic forms, Hg^+^ and Hg^++^, have low lipophilicity and thus a limited ability to cross cell membranes. The mercuric form (Hg^++^) in the bloodstream binds to cysteine sulfhydryl groups (-SH) on erythrocytes, glutathione, and metallothioneines or is transported suspended in plasma [27]. It is mainly absorbed through the respiratory tract, and in small extent through the skin (5%–8%) and gastrointestinal tract (3%–5%) (Figure 1). The main excretory pathways include urine and feces, with a half-life of about two months. In aquatic and soil environments, mercury is primarily present in its mercuric form, including inorganic (e.g., mercuric hydroxide) and organic mercuric compounds, and secondarily as Hg^0^ [28,29]. Mercuric compounds can be found in different states, as mercuric chloride (HgCl_2_, highly toxic and corrosive), mercuric sulfide (HgS, used as a pigment in paints), and mercury fulminate (Hg(CNO)_2_), used as an explosive detonator). Mercuric mercury in the blood stream binds to –SH groups on erythrocytes, glutathione, and metallothioneines or is transported suspended in plasma. There is experimental evidence that this compound is accumulated in the brain through its binding to cysteines [30]. Inorganic mercury, which is derived from industrial release, is biomethylated to methylmercury (MeHg), primarily by sulfate-reducing bacteria [9,10,31], Although only a minor fraction of total mercury is present as MeHg (typically less than 10% and 3% in water and soil/sediment, respectively), the formation of this compound is an important step in mercury cycling. MeHg is easily absorbed overall into the gastrointestinal tract (Figure 1) and is excreted in feces, and to a lesser extent in urine. Organic mercury crosses the blood-brain and placental barriers and can be transmitted to fetus and, through breast milk, babies can assimilate these toxic compounds with resulting bioaccumulation especially by the liver, brain, kidney, and muscles [9]. MeHg bioaccumulate in the food chain from small creatures to larger predatory fish (i.e., swordfish, shark, king mackerel, tilefish) and sea mammals and can reach high concentrations in organisms, in particular in aquatic environments [28]. Large predatory fish and sea mammals can contain methylmercury amounts that are as much as 100,000 times higher than the surrounding water medium. Consequently, populations with high dietary intake of seafood are likely to be subjected to exposure to high levels of mercury that has been reported to harm the brain, lungs, kidneys, the nervous and immune systems, and also the heart and cardiovascular system [32]. Nevertheless, seafood and fish represent an important and great source of proteins, especially for those populations living near seas, lakes, and rivers. Indeed, fish and shellfish contain proteins, as well as long-chain omega-3 polyunsaturated fatty acids (PUFA), including EPA and docosahexaenoic acid (DHA) (Figure 2), and trace elements as selenium, calcium, and magnesium [33]. The presence of mercury was detected in a wide variety of foods including dairy products as pasta, eggs, meats, poultry, and vegetables. However, the level of mercury in these foods is very low compared to the level found in fish.

Methylmercury may also result from methylation of inorganic mercury by microorganisms in the mouth, when mercury vapor is released from amalgam dental fillings [34], and from non-enzymatic methylation, when Vit B12 in the form of methylcobalamin donates a methyl group to mercury [35]. The different mercury forms are interconvertible in vivo; for example, inhaled elemental mercury vapor is absorbed through the mucous membrane of the lungs and is rapidly oxidized to other forms. The organic compounds of mercury have a higher solubility in lipids than the inorganic species, thus they diffuse more easily through the lipid bilayer of biological membranes, increasing their potential toxicity. Mercury absorbed in the body mainly accumulates in the kidneys and brain. The half-life of mercury in the body is about 70 days. 

**Figure 3:**
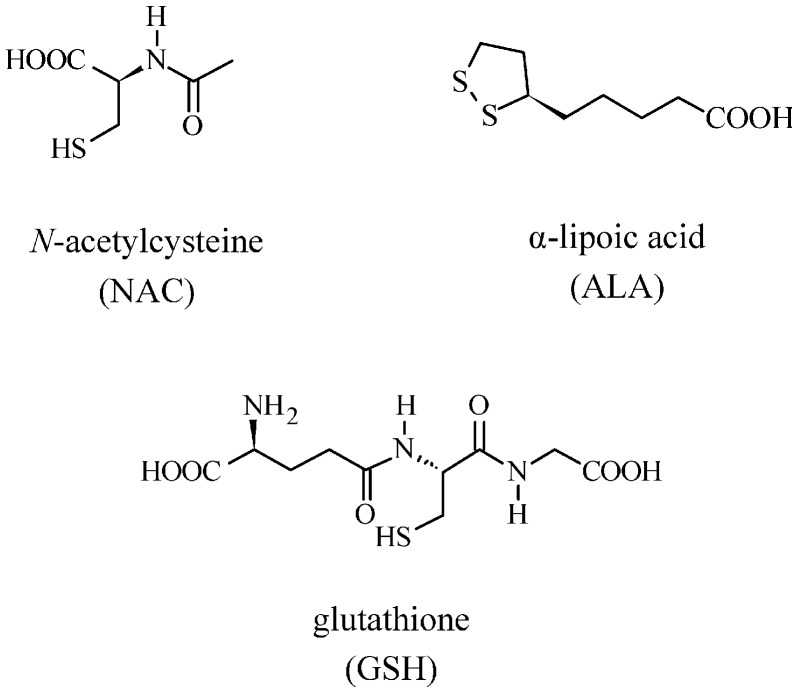
Antioxidants such as *N*-acetylcysteine (NAC), α-lipoic acid (ALA), and glutathione (GSH).

Mercury has no known physiological role in human metabolism; furthermore, the human body lacks effective mechanisms to excrete it [36]. Mercury is not actively excreted by the human body; on average, during the life span of a 70–75 kg human being up to 13 mg of mercury is accumulated in the human body [37]. Mercury is the most dangerous of all heavy metals to which humans and wildlife can be exposed. Both Hg^0^ and MeHg are neurotoxic, whereas inorganic mercury salts are nephrotoxic [38]. Mercury links to numerous biological structures blocking their activity. Indeed, it has a high affinity for sulfhydryl groups (-SH) of aminoacids, proteins, enzymes, and sulfur-containing antioxidants such as *N*-acetylcysteine (NAC), α-lipoic acid (ALA), and glutathione (GSH) (Figure 3). Glutathione provides about 30%–40% of the plasma antioxidant capacity, and is the most potent intracellular and mitochondrial antioxidant for protecting against oxidative stress, inflammation, and cardiovascular diseases [36,37,39,40,41,42].

Indeed, mercury induces oxidative stress and mitochondrial dysfunctions. The latter occur at the NADH (reduced nicotinamide adenine dinucleotide) level: ubiquinone oxidoreductase (complex I of the respiratory chain), cytochrome C, and cytochrome oxidase (complex IV of the respiratory chain), by causing displacement of Fe^2+^ and Cu^+^, by determining depolarization and autoxidation of the inner mitochondrial membrane with a reduction in adenosine 5’-triphosphate (ATP) synthesis, depletion of glutathione, and increased lipid peroxidation [43]. Physiologic consequences include increased hydrogen peroxide, depletion of mitochondrial glutathione, increased lipid peroxidation, oxidation of pyridine nucleotides NAD(P)H (nicotinamide adenine dinucleotide phosphate), and altered calcium homeostasis [43]. Mercury binds to metallothioneines, replacing zinc, copper, and other trace metals, and competes for selenium, reducing the effectiveness of the metalloenzymes. In addition, the complex mercury-selenium reduces the availability of selenium into the formation of the glutathione peroxidase, an enzyme that breaks hydrogen peroxide and other toxic products. Omega-3 polyunsaturated fatty acids of fish and selenium antagonize some of the adverse effects of this heavy metal [44,45,46,47].

## 4. Cardiovascular Effect of Mercury

For many years mercury toxic effects were associated mainly with the central nervous system, kidney and brain; however, mercury may also produce cardiotoxicity [48,49,50,51,52]. It was reported that exposure to mercury compounds, caused by frequent consumption of fish by the population of the Amazon basin (Brazil), has a strong correlation with increased arterial blood pressure (BP) [53,54]. Studies regarding BP and heart rate variability (HRV), among aboriginal populations from Quebec, who are indirectly exposed to mercury and methyl mercury, have been reported by Valera and coauthors [55,56,57,58,59]. These studies suggest a deleterious impact of mercury and MeHg on BP and HRV in Inuit adults, while MeHg exposure during childhood affects HRV among Nunavik Inuit children without concerning BP. Thurston et al. reported that prenatal exposure to MeHg from seafood consumption increases children’s BP [60]. Grandjean et al. studied whether heart function in childhood is affected by exposure to MeHg from seafood. Methylmercury exposure was associated with decreased sympathetic low-frequency and parasympathetic high-frequency modulation of the HRV [61]. Other studies [37,41,62,63] correlate toxic mercury exposure with increased risk of myocardial infarction, atherosclerosis, hypertension, and coronary dysfunction.

Mercury levels in the hair are predictors of the levels of oxidized LDL (low-density lipoprotein) that are frequently found in atherosclerosis lesions and are associated with atherosclerosis disease and acute coronary insufficiency [37,41,64]. The toxic effects of mercury in all its forms have been demonstrated both in vitro, in animals and human beings. Exposure to mercury increases the production of free radicals, reactive oxygen species (ROS), and superoxide anions on account of Fenton reaction [65,66,67,68,69,70]. Mercury binds to thiol (-SH) containing molecules and binds to selenium, forming selenium-mercury complexes, reducing the glutathione peroxidase, catalase, and superoxide dismutase activities due to the absence of selenium in the active site of these enzymes [67,68,70,71,72]. The increment of ROS and the reduction of antioxidant enzymes activity increase the risk of developing cardiovascular disease [73,74]. In addition, mercury increases LDL oxidation and destroys plasma membrane phospholipid integrity by externalization of phosphatidylserine [37,66,75,76]. Moreover, the translocation of phosphatidylserine from the inner to the outer mitochondrial membrane leaflet leads to a modification of mitochondrial membranes with loss of mitochondrial potential and the occurrence of apoptosis. As a consequence, mitochondrial functions are altered, mitochondrial permeability transition (MPT) is affected with a reduction of the membrane potential, oxidative phosphorylation, and ATP production.

Another mechanism through which mercury is responsible for toxic effects on the cardiovascular system is the inactivation of the paraoxonase, an extracellular antioxidative enzyme related to HDL (high-density lipoprotein) [77,78]. The inactivation of paraoxonase causes dysfunctional HDL to reduce reverse cholesterol transport. This enzyme also plays an important role as an antioxidant of LDL, a process that is directly involved in the development of atherosclerosis and in the risk of acute myocardial infarction, cardiovascular disease, coronary heart disease, and carotid artery stenosis [79]. Mercury, in mammals, activates phospholipase A_2_ contributing to the development of several inflammatory diseases correlated with coronary artery disease, acute coronary syndrome, and cerebral plaque rupture [80]. Phospholipase A_2_ catalyzes the hydrolysis of glycerophospholipids at the sn-2 position, producing lysophosphatidic and arachidonic acids. In addition, mercury induces the formation of arachidonic acid metabolites, such as prostaglandins, thromboxanes, leukotrienes, and related compounds that all are considered to be mediators of the inflammatory response even with cardiovascular problems [81]. According to the study by Salonen et al. [37], the level of mercury in the hair and fish intake was positively associated with an increased risk of acute myocardial infarction, and death from cardiovascular disease and coronary heart disease This association was due to the effect of lipid peroxidation catalyzed by mercury that highly contaminated fish in that region.

In Finland, lakes and soil have a low content of selenium, thus Finnish people have low dietary intake of selenium. Mercury effects and selenium deficiency in Eastern Finland populations have been related to myocardial infarction, coronary heart disease and cardiovascular death, lipid peroxidation, and progression of carotid atherosclerosis [82,83]. Fish are rich in selenium, an essential dietary trace element that in human beings is an important component of numerous selenoproteins and selenoenzymes [84,85], and that plays an important role in several antioxidant defense systems, protecting both against cardiovascular diseases and toxic effects of mercury. Indeed, in a population with low selenium intake the toxic effects of mercury may be more pronounced because the metal forms an insoluble complex with selenium, thus reducing its bioavailability in several antioxidant systems (e.g., glutathione peroxidase). Many of the cardiovascular problems related to mercury and mercury compounds are reduced by intake of fish or fish oil, which contain omega-3 PUFA, and by the intake of selenium. Changes in most risk factors are generally evident within weeks as a consequence of fish intake and can result from altered cell membrane fluidity following incorporation of omega-3 PUFA into cell membrane phospholipids [86], as well as direct binding of omega-3 PUFA to cytosolic receptors that regulate gene transcription [87]. Considerable evidence indicates that the consumption of fish reduces coronary heart disease mortality [88], the leading cause of death in developed and in most developing nations. Many cardiovascular problems related to mercury are mitigated by the concomitant intake of fish, which contains omega-3 PUFA, and by intake of selenium [35]. On the other hand, concerns about potential damage from exposure to mercury (present in some fish species) have modified the concept of fish as the model of healthy food. MeHg may be a risk factor for cardiovascular disease, as suggested by mechanistic evidence and experimental animal toxicological studies [89]. However, epidemiological evidence is inconclusive [90]. Roman et al. [13] considered the current epidemiological literature sufficiently robust to support the development of a dose−response function between MeHg exposure and acute myocardial infarction. However, the results of two major cohort studies in the U.S. found no evidence of any clinically relevant adverse effects of Hg exposure on coronary heart disease, stroke, or total CVD disease in adults [91]. The public health implications are potentially significant, as CVD is the leading cause of mortality in most developed countries. Therefore, it is important to improve the characterization of the potential linkage between MeHg exposure and the risk of cardiovascular disease. 

## 5. Mercury Chelating Agents

In acute mercury intoxication, after metal absorption into the circulatory system, to avoid further distribution and penetration in tissues, the elimination of mercury from the body should be envisaged in order to reduce more serious damage. Employing mercury chelating agents [92], inducing diuresis, modulating urinary pH for metal excretion, employing complexing agents to enhance fecal excretion for metals undergoing extensive enterohepatic circulation, and finally hemodialysis, may be employed. The applicability and efficacy of these techniques vary depending upon type, intensity, and extent of exposure and conditions of the patient.

**Figure 4:**
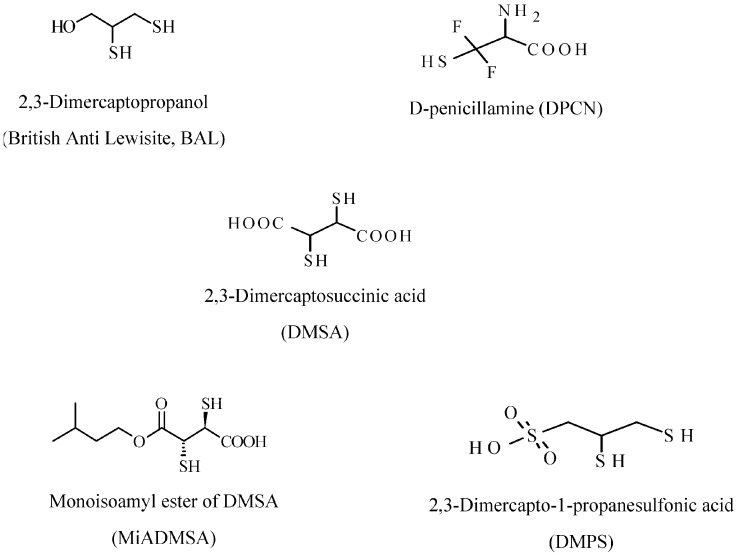
Structures of common substrates chelating mercury.

Chelating agents (Figure 4), that can be used for inorganic mercury poisoning (Hg^0^ and Hg^++^) include: 2,3-dimercaptopropanol (British Anti Lewisite, BAL), d-penicillamine (DPCN), 2,3-dimercaptosuccinic acid (DMSA), monoisoamyl ester of DMSA (MiADMSA), and 2.3-dimercapto-1-propanesulfonic acid (DMPS). These compounds are able to chelate and immobilize mercury, because they contain sulfhydryl groups (-SH). BAL is highly water-soluble and can be administered orally and intravenously. DPCN is a water soluble derivative of penicillin, and increases the excretion of mercury through the urine. This drug is used only for metallic and inorganic mercury poisoning, but it cannot to be used for organic mercury poisoning [93]. Contraindications for this agent include: thrombopenia, proteinuria, hematuresis, and nephrotic syndrome. DMSA increases excretion of methylmercury and inorganic mercury and when this drug is administered orally, its absorption rate is about 20%, which differs from DMPS, whose absorption rate is about 40% when taken orally. DMPS is a more stable form of DMSA, and it is frequently administered intravenously with a half-life of about 20 h. DMPS, as DMSA, promotes the excretion of methylmercury and inorganic mercury in urine. The water soluble MiADMSA is a derivative of DMSA and is administered via oral and intraperitoneal route, although oral administration has been found to be better than intraperitoneal injection. The concomitant administration of DMSA and MiADMSA has demonstrated better results than DMSA or MiADMSA administrated alone, thus allowing for a reduction in the amount of chelating agents, thereby promoting better clinical recovery and minimizing side effects [92].

## 6. Conclusions

Mercury is among the most toxic heavy metals and has no known physiological role in human beings. History has left us with a lot of information and records, regarding the effect of mercury toxicity on the humans. The earliest recorded death caused by mercury is the one of Qin Shi Huang, an Emperor of China (260–210 BC). The existence of different intake pathways of mercury (air, water, food, vaccines, pharmaceuticals, and cosmetics) accounts for its easy accessibility to humans. In particular, in populations whose diet is based mainly on fish consumption, the risk of mercury exposure is increased. In many developing countries, mercury is still a big problem. Efforts have to be made to reduce global mercury use. Exposure to mercury and its compounds has resulted in harmful effects to human health as documented by the monstrous disasters that originated from industrial spills in Japan (Minamata Bay and Agano River) and a rural poisoning in Iraq from MeHg-based fungicide. Mercury has a high affinity for thiol and seleno groups that are present in aminoacids as cysteine and Se-cysteine, *N*-acetylcysteine, lipoic acid, proteins, and enzymes. Cysteine is a precursor for the biosynthesis of glutathione, which is among the most powerful intracellular antioxidants. Mercury and methylmercury induce mitochondrial dysfunction, lowers ATP synthesis, depletes glutathione, and increases phospholipid, protein, and DNA peroxidation. Selenium and fish, rich in omega-3 polyunsaturated fatty acids, antagonize mercury toxicity. The vascular effects of mercury include increases in oxidative stress and inflammation, reductions in oxidative defense, thrombosis, and mitochondrial dysfunction, depolarization, and autoxidation of the inner mitochondrial membrane. Another mechanism through which mercury exerts toxic effects on the cardiovascular system is the inactivation of paraoxonase, which causes dysfunctional HDL to reduce reverse cholesterol transport to the liver. This enzyme plays an important role as an antioxidant of LDL, thus it is directly involved in atherosclerosis, myocardial infarction, and cardiovascular disease. Mercury toxicity is indeed strongly correlated with hypertension, coronary heart disease, myocardial infarction, cardiac arrhythmias, carotid artery obstruction, cerebrovascular accident, and generalized atherosclerosis. Although in populations that eat fish the risk of mercury exposure is increased, important evidence, from human experimental studies indicate that even modest consumption of fish and seafood significantly reduces cardiac disease and death. Selenium and fish rich in omega-3 polyunsaturated fatty acids antagonize mercury toxicity. In any case, it is necessary to elucidate the risks and benefits of fish consumption and balance the toxic effects of mercury with the benefits derived from omega-3 polyunsaturated fatty acids consumption. Mercury toxicity should be evaluated in any patient with hypertension, coronary heart disease, cerebral vascular disease, or other vascular diseases and in patients who have a clinical history of exposure or clinical evidence on examination of mercury overload. The development of a dose-response function relating MeHg exposures with MIs for use in regulatory benefits analyses of future rules targeting Hg air emissions should be envisaged.
